# Supplementary material for: Zero-field quantum beats and spin decoherence mechanisms in CsPbBr3 perovskite nanocrystals
Source: Nat Commun. 2023 Apr 29;14:2472. doi: 10.1038/s41467-023-37721-4 (PMC10148794; doi:10.1038/s41467-023-37721-4)
Supplement: Supplementary file 1 — Supplementary Information [file 41467_2023_37721_MOESM1_ESM.pdf]

Supplementary Information

to

Zero-Field Quantum Beats and Spin Decoherence Mechanisms in $CsPbBr_3$ Perovskite Nanocrystals

Rui Cai[1], Indrajit Wadgaonkar[1], Jia Wei Melvin Lim[1,2], Stefano Dal Forno[1], David Giovanni[1], Minjun Feng[1], Senyun Ye[1], Marco Battiato[1] & Tze Chien Sum[1]

[1]Division of Physics and Applied Physics, School of Physical and Mathematical Sciences, Nanyang Technological University, 21 Nanyang Link, Singapore 637371, Singapore.

[2]ERI@N, Interdisciplinary Graduate School, Nanyang Technological University, 50 Nanyang Avenue, S2-B3a-01, Singapore 639798, Singapore.

Supplementary Note 1 Exciton spin relaxation under optical orientation.

Circularly polarized transient absorption spectroscopy (CTA) is used to study the exciton spin dynamics (Supplementary Fig. 1a). The exciton spin-relaxation time is extracted from the CTA spectrum, with the condition that the excitation density is low enough to avoid any high-order recombination and resonant pumping is guaranteed so that the cooling effect can be excluded. The optical transitions are shown in Supplementary Fig. 1b. Given the exciton spin flip rate W_X, exciton recombination rate γ_X, and the exciton population $N_{\pm 1}$ (The superposition of $|+1\rangle$ and $|-1\rangle$ states and hence the oscillatory part here is assumed to be negligible for simplicity), the rate equations with an initial population G_0 upon a σ^+ pump can be written as

$$\frac{dN_{+1}}{dt} = G_0\delta(t) - (W_X + \gamma_X)N_{+1} + W_X N_{-1} \quad (1)$$

$$\frac{dN_{-1}}{dt} = W_X N_{+1} - (W_X + \gamma_X)N_{-1} \quad (2)$$

solve for $(N_{+1} - N_{-1})$ and then we have the net spin polarization $S_z \propto (N_{+1} - N_{-1}) \propto (\Delta A_{\sigma^+\sigma^+} - \Delta A_{\sigma^+\sigma^-}) \propto e^{-2W_X t}$, with the approximation that $\gamma_X \ll W_X$. Defining the spin-relaxation rate $\frac{1}{\tau_s} = 2W_X$, the spin-relaxation times can be obtained from the CTA spectra. We note that multi-exponential dynamics might be observed due to multiple spin-relaxation channels with different rates, the unequal spin-relaxation rate of electrons and holes in non-excitonic systems, and other spin-selective effects.

Supplementary Note 2 Calculation of the circularly polarized transient absorption spectra.

The optical response of the NCs with the consideration of exciton fine structure splitting (FSS) and CTA signal follow the treatment for a CdSe/ZnSe quantum dot[1]. In a single $CsPbBr_3$ NC, we consider an orthorhombic distortion of D_{2h} symmetry which splits the $J = 1$ exciton states into three non-degenerate states, that are linearly polarized along the three symmetry axes respectively. Coherently excite superposition of any in-plane dipoles (here in-plane means the substrate plane that is perpendicular to the laser beam direction) allows to observe the quantum beats (either by linearly polarized pump that is not perfectly aligned to one dipole only, or by circularly polarized pump that inject spins at the same time). Regardless of the size distribution and tilted orientations, the manifested quantum beats measure the average splitting energies between any two FSS levels due to the random orientations.

The dynamics of the quantum system is initially calculated with an external potential from the in-coming light fields via a Liouville equation. Then the probe field change (and hence the TA signal) that is induced by the polarization field originating from the dynamics of the excited states, is calculated by the macroscopic Maxwell equations.

The optically induced dynamics of the system with optical stimulus and the relaxation processes can be described by the quantum mechanical Liouville equation with Lindblad dissipator, and the density matrix of the interaction between two states *a* and *b* can be approximated as

$$\dot{\rho}_{a,b}(t) = -i\frac{1}{\hbar}([\hat{H}^0 + \hat{H}^\gamma(t), \hat{\rho}(t)])_{a,b} + D_{a,b}(\hat{\rho}) \tag{3}$$

where $\hat{H}^0$ and $\hat{H}^\gamma(t)$ refer to the stationary energies of levels and the influence of the optical fields, and $D_{a,b}(\hat{\rho})$ the Lindblad dissipator. The dynamics starts with full occupation of the exciton ground state and are separated temporally.

Dynamics during the laser pulses

The light-matter interaction can be described with the typical dipole transitions and rotating wave approximation (RWA) that

$$\hat{H}^\gamma(t) = -\mathbf{E}_{in}^+(t)\bullet\hat{\mathbf{d}}^+ - \mathbf{E}_{in}^-(t)\bullet\hat{\mathbf{d}} \tag{4}$$

with the dipole operators $\hat{\mathbf{d}}^+$ and $\hat{\mathbf{d}}$ describing the excitation and relaxation respectively, and the electric field $\mathbf{E}_{in}^\pm = \mathbf{E}_{pump}^\pm + \mathbf{E}_{probe}^\pm$, where

$$\mathbf{E}_{pu/pr}^+(t) = (k_x^{pu/pr}\mathbf{e}_x + k_y^{pu/pr}\mathbf{e}_y)E_{pu/pr}^0(t)e^{-i(\omega_{pu/pr}t+\varphi_{pu/pr})} \tag{5}$$

$$\mathbf{E}_{pu/pr}^-(t) = (k_x^{pu/pr}\mathbf{e}_x + k_y^{pu/pr}\mathbf{e}_y)E_{pu/pr}^{0*}(t)e^{i(\omega_{pu/pr}t+\varphi_{pu/pr})} \tag{6}$$

with defined light polarization $k_x^{pu/pr}\mathbf{e}_x + k_y^{pu/pr}\mathbf{e}_y$ ($k_x^{pu/pr} = 0$ for horizontal polarization and $k_y^{pu/pr} = 0$ for vertical polarization, and $k_{pu/pr}(\mathbf{e}_x \pm i\mathbf{e}_y)$ for circular polarization).

The relaxation and dephasing are neglected to calculate the dynamics during short laser pulses, within the interaction picture $\tilde{\rho}_{a,b}(t) = e^{i\frac{1}{\hbar}(H_{a,a}^0 - H_{b,b}^0)t}\rho_{a,b}(t)$ and then the remaining equation of motion

$$\dot{\tilde{\rho}}_{a,b}(t) = -i\frac{1}{\hbar}\sum_j\left(\tilde{H}_{a,j}^{\gamma,pu/pr}(t)\tilde{\rho}_{j,b}(t) - \tilde{\rho}_{a,j}(t)\tilde{H}_{j,b}^{\gamma,pu/pr}(t)\right) \tag{7}$$

where

$$\tilde{H}_{j,b}^{\gamma,pu/pr}(t) = H_{j,b}^{\gamma,pu/pr}(t)e^{i\omega_{j,b}t} = -E_{pu/pr}^0(t)e^{-i(\varphi_{pu/pr}+\omega_{j,b}t)}\vec{J}\bullet\vec{d}_{j,b}^+ + h.c. \tag{8}$$

with *h.c.* the Hermitian adjoint term. With an approximation of the jump condition, the effect on the density matrix by the pulse can be described as

$$\hat{\rho}^{\text{after}\, pu/pr}(t) = e^{-i\frac{1}{\hbar}\hat{\Lambda}^{\gamma,pu/pr}} \hat{\rho}^{\text{before}\, pu/pr} \left(e^{-i\frac{1}{\hbar}\hat{\Lambda}^{\gamma,pu/pr}} \right)^{+} \tag{9}$$

in which

$$\Lambda_{j,b}^{\gamma,pu/pr} = -\int E_{pu/pr}^{0}(t)dt \cdot e^{-i\varphi_{pu/pr}} (k_x^{pu/pr}\mathbf{e}_x + k_y^{pu/pr}\mathbf{e}_y)\bullet\mathbf{d}_{j,b}^{+}\tilde{\delta}_{\omega_{pu/pr},\omega_{j,b}} + h.c. \tag{10}$$

with $\tilde{\delta}_{\omega_{pump},\omega_{x/y,GS}} = 1$ stands for the transition between the ground state and the *x*/*y* state upon pump pulse and $\tilde{\delta}_{\omega_{probe},\omega_{XX,x/y}} = 1$ between the *x*/*y* state and the biexciton state upon probe pulse respectively, and $\tilde{\delta}_{\omega_{pu/pr},\omega_{j,b}} = 0$ for other *j* values. For perfectly linearly polarized transitions $\langle a|\mathbf{d}|b\rangle = d_0\mathbf{e}_{x/y}$, the non-vanishing matrix elements of $\hat{\Lambda}^{\gamma,pu/pr}$ writes

$$\hat{\Lambda}^{\gamma,pu/pr} = -\int E_{pu/pr}^{0}(t)dt \cdot \begin{pmatrix} GS & y & x & XX \\ 0 & e^{i\varphi_{pu/pr}}\mu_y^{*} & e^{i\varphi_{pu/pr}}\mu_x^{*} & 0 \\ e^{-i\varphi_{pu/pr}}\mu_y & 0 & 0 & e^{i\varphi_{pu/pr}}v_y^{*} \\ e^{-i\varphi_{pu/pr}}\mu_x & 0 & 0 & e^{i\varphi_{pu/pr}}v_x^{*} \\ 0 & e^{-i\varphi_{pu/pr}}v_y & e^{-i\varphi_{pu/pr}}v_x & 0 \end{pmatrix} \tag{10}$$

with $\mu_{x/y} = k_{pu}^{x/y} d_0$ and $v_{x/y} = k_{pr}^{x/y} d_0$ (For simplicity, we use d_0 for both *x* and *y* states).

TA signal detection: dynamics between and after the pump and probe pulses

The contributions from the Lindblad dissipator must be considered to calculate the dynamics between the pulses, which can be separated into the relaxation processes and pure dephasing

$$\hat{D}(\hat{\rho}) = \hat{D}^{relax.}(\hat{\rho}) + \hat{D}^{deph.}(\hat{\rho}) \tag{11}$$

Since the exciton population time is much longer than the oscillating period, the relaxation between *x* and *y* states can be neglected considering the time averaging. Therefore, the relaxation processes can be described by $D_{x/y,x/y}^{relax.} = -\gamma_{x/y,x/y}\rho_{x/y,x/y}$ and $D_{GS,GS}^{relax.} = \gamma_{x,x}\rho_{x,x} + \gamma_{y,y}\rho_{y,y}$, with defined $\gamma_{x,x} = \gamma_{y,y} = \frac{1}{\tau_x} = \frac{1}{\tau_y} = \frac{1}{\tau_r}$ (where $\tau_x = \tau_y = \tau_r$ means

the equal exciton population lifetime for x and y states). The dephasing terms consist of the quantum beats dephasing $D_{x,y}^{deph.} = -\frac{1}{\tau_{xy}}\rho_{x,y}$, and the interband pure dephasing $D_{GS,x/y}^{deph.} = -\frac{1}{\tau_{\delta_{GS,x/y}}}\rho_{GS,x/y}$ and $D_{x/y,XX}^{deph.} = -\frac{1}{\tau_{\delta_{x/y,XX}}}\rho_{x/y,XX}$ where $\tau_{\delta_{a,b}}$ refers to the pure dephasing time.

Between the pump and probe pulses, the term $\hat{H}^{\gamma}(t)$ in Eq. (3) vanishes and only the free evolution due to $\hat{H}^{0}$ and $D_{a,b}(\hat{\rho})$ are considered. For the remaining dephasing and relaxation processes, an analytical solution can be given in the block-diagonal form

$$\hat{\rho}(t) = \begin{pmatrix} 0 & 0 \\ 0 & \hat{\rho}_{measure}(t) \end{pmatrix} \tag{12}$$

where

$$\hat{\rho}_{measure}(t) = \left\{ \begin{array}{cccc} GS & y & x & XX \\ 1-\rho_{y,y}(t_0)e^{-\frac{t}{\tau_r}} - \rho_{x,x}(t_0)e^{-\frac{t}{\tau_r}} & \rho_{GS,y}(t_0)e^{i\omega_{y,GS}t}e^{-\frac{t}{\tau_{\delta y,GS}}} & \rho_{GS,x}(t_0)e^{i\omega_{x,GS}t}e^{-\frac{t}{\tau_{\delta x,GS}}} & 0 \\ \rho_{y,GS}(t_0)e^{-i\omega_{y,GS}t}e^{-\frac{t}{\tau_{\delta y,GS}}} & \rho_{y,y}(t_0)e^{-\frac{t}{\tau_r}} & \rho_{y,x}(t_0)e^{i\omega_{xy}t}e^{-\frac{t}{\tau_{xy}}} & \rho_{y,XX}(t_0)e^{i\omega_{y,XX}t}e^{-\frac{t}{\tau_{\delta y,XX}}} \\ \rho_{x,GS}(t_0)e^{-i\omega_{x,GS}t}e^{-\frac{t}{\tau_{\delta x,GS}}} & \rho_{x,y}(t_0)e^{-i\omega_{xy}t}e^{-\frac{t}{\tau_{xy}}} & \rho_{x,x}(t_0)e^{-\frac{t}{\tau_r}} & \rho_{x,XX}(t_0)e^{i\omega_{x,XX}t}e^{-\frac{t}{\tau_{\delta x,XX}}} \\ 0 & \rho_{XX,y}(t_0)e^{-i\omega_{y,XX}t}e^{-\frac{t}{\tau_{\delta y,XX}}} & \rho_{XX,x}(t_0)e^{-i\omega_{x,XX}t}e^{-\frac{t}{\tau_{\delta x,XX}}} & \rho_{XX,XX}(t_0) \end{array} \right\} \tag{13}$$

with the respective previous laser pulse time t_0.

The induced polarization can then be calculated via $\mathbf{P}(t) = Tr(\hat{\mathbf{d}}\hat{\rho}(t))$, with which the signal field out from the NC $\mathbf{E}_{out}(t)$ can be calculated by the macroscopic Maxwell equations that

$$\mathbf{E}_{out}(t) = \mathbf{E}_{in}(t) + i\frac{\omega_c \mu_0 c l}{2}\mathbf{P}(t) \tag{14}$$

where ω_c, μ_0, c, l denote the laser frequency, vacuum permeability, the speed of light, and the NC edge length along z-axis.

The intensity of the signal field in frequency domain is then

$$I_{out}(\omega) \sim |\mathbf{E}_{out}(\omega)|^2 = |\mathbf{E}_{in}(\omega)|^2 + \omega_c \mu_0 cl \,\mathrm{Im}(\mathbf{E}_{in}(\omega)\mathbf{P}^*(\omega)) + \frac{1}{4}|\omega_c \mu_0 cl \mathbf{P}(\omega)|^2 \quad (15)$$

The heterodyne detection allows a good approximation of the TA signal that[2]

$$\Delta A(\omega) \sim -\omega_c l \,\mathrm{Im}[\mathbf{E}_{in}(\omega)\mathbf{P}^*(\omega)] \quad (16)$$

The signal around the biexciton resonances (XX) can be calculated by

$$\Delta A_{x/y}^{XX}(\omega) \sim \mathrm{Im}[\mathbf{E}_{in}(\omega)\mathbf{P}_{x/y}^{*XX}(\omega)] \quad (17)$$

where $\mathbf{P}^{*XX}_{x/y}(\omega) = \mathcal{F}(\rho_{XX,x/y}(t) d_0 \mathbf{e}_{x/y})(\omega)$ by Fourier transform.

For a given time delay t_D between the pump and probe pulses, the solution gives the TA signal for the *y* dipole that

$$\begin{aligned}
\Delta A_y^{XX}(\omega, t_D) \sim & -\frac{\sin(\frac{A_{pu}^0}{2\hbar}\sqrt{|\mu_x|^2+|\mu_y|^2})^2 \sin(\frac{A_{pr}^0}{2\hbar}\sqrt{|\nu_x|^2+|\nu_y|^2})}{(|\mu_x|^2+|\mu_y|^2)(|\nu_x|^2+|\nu_y|^2)^{\frac{3}{2}}} \times \\
& \{\frac{-\frac{1}{\tau_{\delta_{y,XX}}}}{\frac{1}{\tau_{\delta^2_{y,XX}}}+(\omega_{y,XX}-\omega)^2}[(|\nu_y|^2|\nu_x|^2(|\mu_y|^2-|\mu_x|^2)+|\nu_y|^2\cos(\frac{A_{pr}^0}{2\hbar}\sqrt{|\nu_x|^2+|\nu_y|^2}(|\mu_y|^2|\nu_y|^2+|\mu_x|^2|\nu_x|^2)))e^{-\frac{t_D}{\tau_r}} + \\
& |\nu_y|^2\cos(\frac{A_{pr}^0}{2\hbar}\sqrt{|\nu_x|^2+|\nu_y|^2})(\mu_y^*\mu_x\nu_y^*\nu_x e^{i\omega_{xy}t_D} + \mu_y\mu_x^*\nu_y\nu_x^* e^{-i\omega_{xy}t_D})e^{-\frac{t_D}{\tau_{xy}}} + \\
& \frac{|\nu_x|^2-|\nu_y|^2}{2}(\mu_y^*\mu_x\nu_y^*\nu_x e^{i\omega_{xy}t_D} + \mu_y\mu_x^*\nu_y\nu_x^* e^{-i\omega_{xy}t_D})e^{-\frac{t_D}{\tau_{xy}}}] + \\
& \frac{(\omega_{y,XX}-\omega)}{\frac{1}{\tau_{\delta^2_{y,XX}}}+(\omega_{y,XX}-\omega)^2}[\frac{|\nu_x|^2+|\nu_y|^2}{2i}(\mu_y^*\mu_x\nu_y^*\nu_x e^{i\omega_{xy}t_D} - \mu_y\mu_x^*\nu_y\nu_x^* e^{-i\omega_{xy}t_D})e^{-\frac{t_D}{\tau_{xy}}}]\}
\end{aligned} \quad (18)$$

and for the *x* dipole,

$$\Delta A_x^{XX}(\omega,t_D)\sim-\frac{\sin(\frac{A_{pu}^0}{2\hbar}\sqrt{|\mu_x|^2+|\mu_y|^2})^2\sin(\frac{A_{pr}^0}{2\hbar}\sqrt{|\nu_x|^2+|\nu_y|^2})}{(|\mu_x|^2+|\mu_y|^2)(|\nu_x|^2+|\nu_y|^2)^{\frac{3}{2}}}\times$$

$$\begin{aligned}
&\{\frac{-\frac{1}{\tau_{\delta_{x,XX}}}}{\frac{1}{\tau_{\delta^2_{x,XX}}}+(\omega_{x,XX}-\omega)^2}[(|\nu_y|^2|\nu_x|^2(|\mu_x|^2-|\mu_y|^2)+|\nu_x|^2\cos(\frac{A_{pr}^0}{2\hbar}\sqrt{|\nu_x|^2+|\nu_y|^2}(|\mu_y|^2|\nu_y|^2+|\mu_x|^2|\nu_x|^2)))e^{-\frac{t_D}{\tau_r}}+\\
&|\nu_x|^2\cos(\frac{A_{pr}^0}{2\hbar}\sqrt{|\nu_x|^2+|\nu_y|^2})(\mu_y^*\mu_x\nu_y^*\nu_x e^{i\omega_{xy}t_D}+\mu_y\mu_x^*\nu_y\nu_x^* e^{-i\omega_{xy}t_D})e^{-\frac{t_D}{\tau_{xy}}}+\\
&\frac{|\nu_y|^2-|\nu_x|^2}{2}(\mu_y^*\mu_x\nu_y^*\nu_x e^{i\omega_{xy}t_D}+\mu_y\mu_x^*\nu_y\nu_x^* e^{-i\omega_{xy}t_D})e^{-\frac{t_D}{\tau_{xy}}}]-\\
&\frac{(\omega_{x,XX}-\omega)}{\frac{1}{\tau_{\delta^2_{x,XX}}}+(\omega_{x,XX}-\omega)^2}[\frac{|\nu_x|^2+|\nu_y|^2}{2i}(\mu_y^*\mu_x\nu_y^*\nu_x e^{i\omega_{xy}t_D}-\mu_y\mu_x^*\nu_y\nu_x^* e^{-i\omega_{xy}t_D})e^{-\frac{t_D}{\tau_{xy}}}]\}
\end{aligned} \tag{19}$$

in which $A^0_{pu/pr}=\int E^0_{pu/pr}(t)dt$. Given that $\mu_x=\mu_y$, $\nu_x=\nu_y$ (with $k_x^{pu/pr}=k_y^{pu/pr}$), and $\mu_y^*\mu_x\nu_y^*\nu_x=|\mu|^2|\nu|^2e^{i\varphi}$ with φ being the relative phase between the excitation and readout, and $\Gamma_{deph.\_XX}=\frac{1}{\tau_{\delta_x,XX}}=\frac{1}{\tau_{\delta_y,XX}}$ defining the linewidth, the TA signal reads that

$$\Delta A_{x/y}^{XX}(\omega,t_{\mathrm{D}})\sim \frac{\Gamma_{deph.\_XX}}{\Gamma^2_{deph.\_XX}+(\omega_{x/y,XX}-\omega)^2}\left(e^{-\frac{t_{\mathrm{D}}}{\tau_r}}+e^{-\frac{t_{\mathrm{D}}}{\tau_{xy}}}\cos(\omega_{xy}t_{\mathrm{D}}+\varphi)\right)\pm\frac{(\omega_{x/y,XX}-\omega)}{\Gamma^2_{deph.\_XX}+(\omega_{x/y,XX}-\omega)^2}e^{-\frac{t_{\mathrm{D}}}{\tau_{xy}}}\sin(\omega_{xy}t_{\mathrm{D}}+\varphi) \tag{20}$$

Similarly, the TA signal around the exciton resonance can be obtained as

$$\Delta A_{x/y}^{X}(\omega,t_{\mathrm{D}})\sim -\frac{\Gamma_{deph.\_X}}{\Gamma^2_{deph.\_X}+(\omega_{GS,x/y}-\omega)^2}\left(e^{-\frac{t_{\mathrm{D}}}{\tau_r}}+e^{-\frac{t_{\mathrm{D}}}{\tau_{xy}}}\cos(\omega_{xy}t_{\mathrm{D}}+\varphi)\right)\mp\frac{(\omega_{GS,x/y}-\omega)}{\Gamma^2_{deph.\_X}+(\omega_{GS,x/y}-\omega)^2}e^{-\frac{t_{\mathrm{D}}}{\tau_{xy}}}\sin(\omega_{xy}t_{\mathrm{D}}+\varphi) \tag{21}$$

As for the dynamics around the photoinduced absorption (PIA), we empirically write (because it shares the same excited states)

$$\Delta A_{x/y}^{PIA}(\omega,t_{\mathrm{D}})\sim \frac{\Gamma_{deph.\_PIA}}{\Gamma^2_{deph.\_PIA}+(\omega_{x/y,PIA}-\omega)^2}\left(e^{-\frac{t_{\mathrm{D}}}{\tau_r}}-e^{-\frac{t_{\mathrm{D}}}{\tau_{xy}}}\cos(\omega_{xy}t_{\mathrm{D}}+\varphi)\right)\mp\frac{(\omega_{x/y,PIA}-\omega)}{\Gamma^2_{deph.\_\mathrm{PIA}}+(\omega_{x/y,PIA}-\omega)^2}e^{-\frac{t_{\mathrm{D}}}{\tau_{xy}}}\sin(\omega_{xy}t_{\mathrm{D}}+\varphi) \tag{22}$$

Eq. (20)~(22) can be phenomenologically understood as periodic temporal modulation of absorption lines with a Lorentzian profile. When the probe energy is tuned to the X/XX/PIA resonance, the last term describing the dispersive contribution vanishes and the temporal profile of CTA signal can be described by a damped cosine function. The total CTA signal can be estimated by

$$\Delta A \sim \Delta A_x^{\mathrm{X}} + \Delta A_y^{\mathrm{X}} + \Delta A_x^{\mathrm{XX}} + \Delta A_y^{\mathrm{XX}} + \Delta A_x^{\mathrm{PIA}} + \Delta A_y^{\mathrm{PIA}} \tag{23}$$

Eq. (23) is used to calculate the CTA spectra shown in Fig. 1d~f.

Set the probing frequency ω to the exciton resonance ($\omega = \omega_{GS,x}, \omega = \omega_{GS,y}$), around the exciton resonance we have

$$S_z \propto (\Delta A_{\sigma^+\sigma^+} - \Delta A_{\sigma^+\sigma^-}) =$$
$$[\Delta A_x^X(\omega_{GS,x}, t_D, \varphi = 0) + \Delta A_y^X(\omega_{GS,y}, t_D, \varphi = 0)] - [\Delta A_x^X(\omega_{GS,x}, t_D, \varphi = \pi) + \Delta A_y^X(\omega_{GS,y}, t_D, \varphi = \pi)] \sim$$
$$-\frac{4}{\Gamma_{deph.\_X}} e^{-\frac{t_D}{\tau_{xy}}} \cos \omega_{xy} t_D$$

(24)

Set ω to the biexciton resonance ($\omega = \omega_{x,XX}, \omega = \omega_{y,XX}$), we have the similar result that (because of the selection rule, $\varphi = \pi$ for $\Delta A_{\sigma^+\sigma^+}$ and $\varphi = 0$ for $\Delta A_{\sigma^+\sigma^-}$)

$$S_z \propto (\Delta A_{\sigma^+\sigma^+} - \Delta A_{\sigma^+\sigma^-}) =$$
$$[\Delta A_x^{XX}(\omega_{x,XX}, t_D, \varphi = \pi) + \Delta A_y^{XX}(\omega_{y,XX}, t_D, \varphi = \pi)] - [\Delta A_x^{XX}(\omega_{x,XX}, t_D, \varphi = 0) + \Delta A_y^{XX}(\omega_{y,XX}, t_D, \varphi = 0)] \sim$$
$$-\frac{4}{\Gamma_{deph.\_XX}} e^{-\frac{t_D}{\tau_{xy}}} \cos \omega_{xy} t_D$$

(25)

From which the beating frequency ω_{xy} can be obtained.

Supplementary Note 3 The Elliott-Yafet (EY) process in $CsPbBr_3$ polycrystalline thin film.

The EY mechanism is suggested to be inefficient for conduction band electrons in wide-gap III-V semiconductors owing to the rather weak SOC and large band gap which reduces spin-mixing strength[3]. A weak EY process might also be expected in LHPs. For both valence and conduction bands, the bandgap reduces the spin-mixing strength due to the inverted band structure in LHPs. It has been shown that the EY mechanism is responsible for spin depolarization of free carriers in $CsPbI_3$ NCs by absorbing/emitting longitudinal optical (LO) phonons[4]. Indeed, the strong carrier-phonon interaction in LHPs contributes significantly to the momentum scattering events as revealed by temperature-dependent measurements[5-7]. However, weak temperature dependences of spin-relaxation times have been observed in three-dimensional (3D) $CsPbBr_3$, where the Bir-Aronov-Pikus (BAP)[8] and the D'yakonov-Perel' (DP)[9] mechanisms have been claimed. Here we show that the EY process exists in 3D $CsPbBr_3$ polycrystalline thin film (PTF). Supplementary Fig. 2a displays the temperature-dependent net spin polarization of $CsPbBr_3$ PTF with excitations near the band edge and with the same population density for all temperatures, and we note that $CsPbBr_3$ does not undergo any significant structural phase transition in this temperature range[10]. The results show a tendency that the spin-relaxation time decreases with temperature which indicates a thermally enhanced spin-relaxation rate. Nevertheless, these dynamics cannot be well-fitted by single-exponential decays. By introducing a temperature-independent component τ_1 which we attribute to the spin-selective optical Stark effect[11], we fit the traces with a thermal contribution τ_2.

We evaluate the temperature dependence of τ_2 that is influenced by the phonon-assisted

process with the spin relaxation related, in the range of 80 K to RT. Assuming that the interactions with phonon contribute mostly to the momentum scattering τ_p, which can then be estimated by the homogeneous broadening of photoluminescence (PL) linewidth $\Gamma(T) = \frac{\hbar}{2\tau_p(T)}$. Clearly, as depicted in Supplementary Fig. 2c, the spin lifetime scales linearly with the momentum scattering time, *i.e.*, $\tau_2 \sim \tau_p$ except for an abrupt change around 120 K which is in line with the evolution of the first exciton peak intensity in the absorption spectra (Supplementary Fig. 2d) and the temperature-dependent optical response for a $CsPbBr_3$ single-crystal[12]. Such a variation of electronic structure might change the spin lifetime by the spin-mixing strength. The linear relationship between τ_2 and τ_p falls into the category of EY mechanism for metallic and degenerate semiconductors[13]. While for nondegenerate semiconductors, there is an additional spin-mixing term that is related to the kinetic energy of carriers and depends on temperature quadratically[13, 14]. Here, the direct proportionality between the spin-relaxation rate and momentum scattering rate in $CsPbBr_3$ can be understood by self-doping in LHPs which modifies the Fermi level and make it degenerately doped by resident carriers[15, 16]. Such unintentional self-doping process might occur during the crystallization process because of excess precursors, or be induced by photodoping[17]. Therefore, different fabrication processes take effect on the temperature dependence of spin lifetime as reported[8, 9]. Consequently, we assign the EY process as the dominant mechanism for spin relaxation in $CsPbBr_3$ PTF.

Supplementary Note 4 The effect of pump energy at room temperature (RT).

Upon non-resonant excitation above the band edge, the cooling process cannot be excluded which changes the initial spin polarization degree at the band edge that can be defined as

$$P = \frac{\Delta A_{\sigma^+\sigma^+} - \Delta A_{\sigma^+\sigma^-}}{\Delta A_{\sigma^+\sigma^+} + \Delta A_{\sigma^+\sigma^-}} \times 100\% \tag{26}$$

which represents the difference between co-circular and counter-circular probing signals divided by the sum of them. In polycrystalline thin film, the electron excess energy contributes to spin flip mainly by emitting LO phonons via EY process. In NCs, high excess energy gives large exciton momentum K which flips spins via EY or/and motional narrowing process when cooling towards the band edge. Therefore, high excitation energy far from the band edge results in low initial spin polarization degree. In Supplementary Fig. 3a, the rise time of CTA signal increases with the energy difference between pump and probe energies (ΔE) which evidences the cooling process. Given the scattering events related spin-flip probability α, the initial spin polarization degree P as a function of ΔE can be fitted with

$$P = P_0 e^{-\alpha \Delta E} \tag{27}$$

as shown in Supplementary Fig. 3b.

Supplementary Note 5 Theoretical modelling of the spin relaxation.

We describe an ensemble of spins undergoing three concurrent processes: EY spin flip scatterings, precession of the exciton's spin around an effective magnetic field (representing the quantum beating between the fine-structure splitted Π_x and Π_y states in Fig. 2g) and directed along the centre of mass exciton's momentum, and momentum randomizing scatterings.

We assume that EY scatterings have a lifetime of τ_{EY}, and that they fully randomize the 3D spin direction. The spin precession happens around an effective axis which lies always in the x-y plane. The length of the axis Ω is representative of the FSS of the levels and controls the precession period. We further assume that, with a scattering lifetime τ_{p}, the exciton's centre of mass momentum can be randomized, leading to a randomization of the direction of the effective magnetic field.

For a given set of parameters (τ_{EY}, Ω, and τ_{p}), the spin dynamics is computed by running Monte Carlo simulations of 2000 excitons each characterised by a 3-dimensional spin vector and an effective magnetic field (directed along center of mass (CoM) momentum). Precession of the spin along the effective magnetic field for each exciton is continuously simulated, while EY scatterings (which completely randomise the spin direction) or momentum scatterings (which randomise the direction of the in-plane effective magnetic field) are included as discrete events with the appropriate probability. The ensemble average of the spin projection along the z axis is then extracted and used for fitting with the experimental values.

Supplementary Note 6 Fitting of experimental results and parametrization of the processes.

The fitting with the experimental results is done individually for each temperature and NC's size. This is done by minimizing the squared difference between the theoretical dynamics and the experimental one. The minimization is done using Powell minimization.

The EY scattering lifetimes τ_{EY} are assumed to be independent on the NC's size. This choice was done to reproduce the high temperature behaviour, which shows (except below the temperature at which the motional narrowing process overtakes EY) same dynamics for all the NC's sizes. EY lifetimes τ_{EY} are however left to be temperature dependent. Such temperature dependence is extracted by fitting experimental results for NC12 for temperatures above 210K. Below that tempereature the similar or much smaller efficiency of the EY compared to motional narrowing process in the spin dephasing, prevents an effective fitting. The EY lifetimes τ_{EY} are therefore extrapolated using a $T^{-1.3}$ slope. This is consistent with the results for bulk crystal, where the $T^{-1.3}$ behaviour is kept until well below 200K, where EY will be irrelevant to the spin dynamics in NCs.

We then move onto the fitting of the lowest temperatures, where beatings can be clearly observed. We fit the experimental results allowing for a temperature and size dependent Ω, and τ_p. We find that Ω is somehow size dependent, but clearly not temperature dependent, confirming our interpretation of the results. At intermediate temperatures, it is not anymore possible to fit independently Ω, and τ_p (due to absence of beating signatures, the fitting for Ω,

and τ_p become ill conditioned). We therefore extrapolate in temperature the low temperature values for Ω, and for for τ_p only.

All the results above are summarized in Fig. 3c and 3d in the Main text. The reader can notice that at temperatures above 300 K, the τ_p seem to show an anomalous behaviour. This is however purely the result of the impossibility of the fitting procedure to obtain reliable τ_p for those temperatures. The dynamics in that energy range is so strongly EY dominated that extracting τ_p leads to huge fitting errors. Therefore fitted τ_p above 300 K are not to be interpreted as reliable results and are included only for completeness.

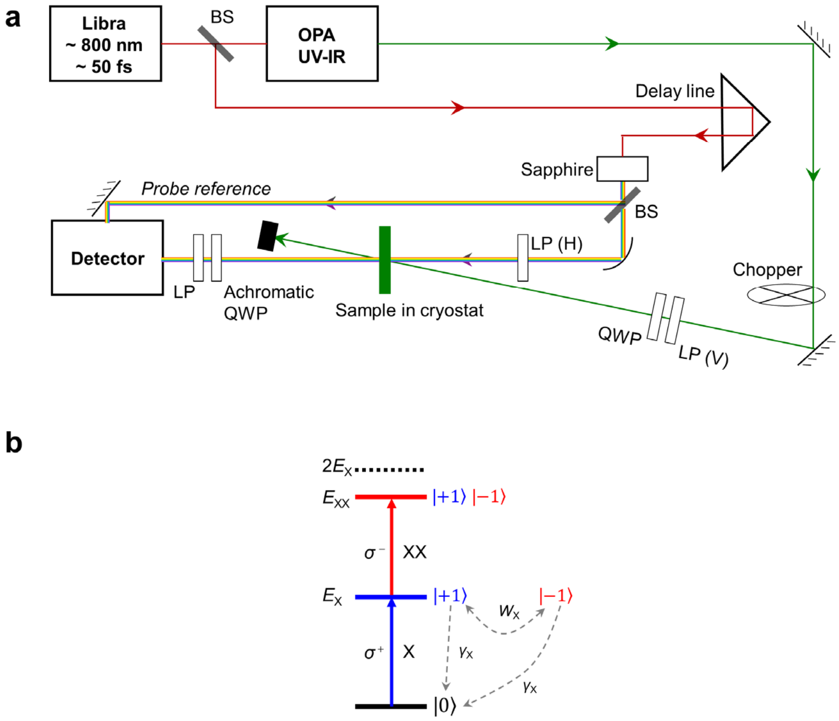

Supplementary Figure 1 a Schematic of the CTA spectroscopy. LP and QWP are short for linear polarizer and quarter wave-plate, respectively. **b** Schematic of the optical transitions and population dynamics of excitons. Here γ_X and W_X represent the direct exciton spin-flip and recombination rates, respectively. E_X and E_{XX} refer to the energy levels of exciton and biexciton, respectively. X and XX refer to the exciton and biexciton transitions, respectively.

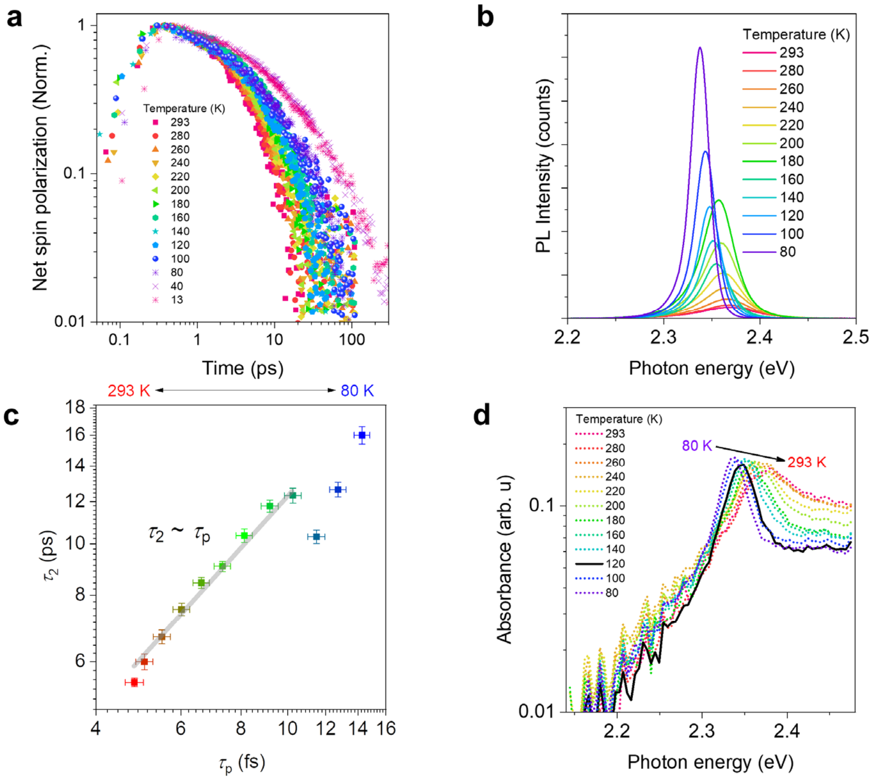

Supplementary Figure 2 Temperature dependence of spin-relaxation time in $CsPbBr_3$ PTF. a Net spin polarization obtained from CTA spectra at different temperatures. The carrier density at 293 K is estimated to be 2.8×10^{16} cm^{-3}. The decays are fitted with a bi-exponential model with fixed τ_1. **b** Temperature-dependent photoluminescence spectra of $CsPbBr_3$ PTF upon a 3.1 eV pulse excitation with a fluence of 0.056 μJ cm^{-2}, which is reduced to 0.028 μJ cm^{-2} below 200 K. **c** The relationship between momentum scattering time and spin-relaxation time τ_2. A grey guide line with exponent of 1 is plotted. **d** Temperature-dependent absorption spectra of $CsPbBr_3$ PTF.

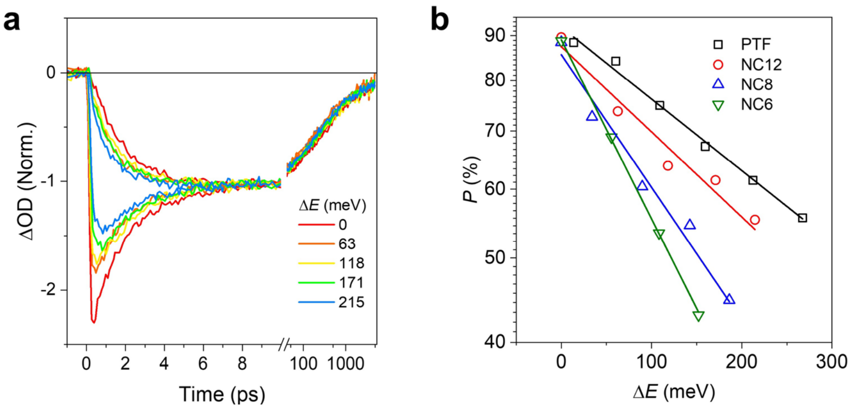

Supplementary Figure 3 The effect of excitation energy on initial spin polarization degree at RT. a Representative CTA spectra for NC12 upon different excitation energies. **b** The initial spin polarization degree at the band edge as a function of the energy difference between pump and probe energy.

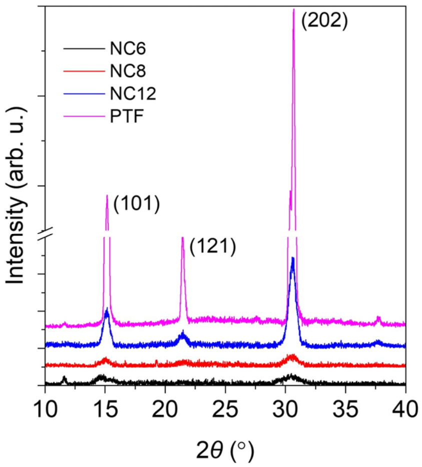

Supplementary Figure 4 X-ray diffraction patterns for $CsPbBr_3$ NCs and PTF. The XRD data were acquired with a Rigaku SmartLab X-ray diffractometer, which uses a Cu Kα radiation source (with a wavelength of 1.542 Å). The XRD results for all samples (no data for NC5 because the poor film quality) show three main peaks located round 15°, 21°, and 31° corresponds to the diffractions from (101), (121), (202) lattice planes in a orthorhombic phase[18].

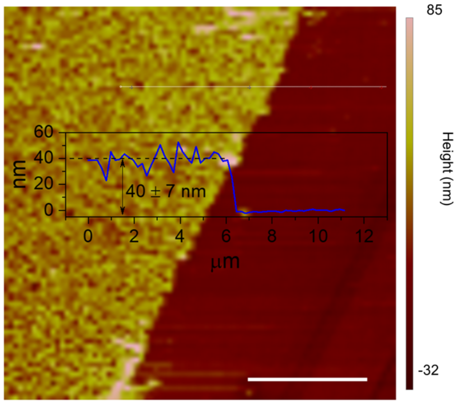

Supplementary Figure 5 The morphology of as-fabricated $CsPbBr_3$ PTF is studied by atomic force microscopy (AFM) using a commercial Bruker Nanoscope AFM in contact mode in air. The step profile indicates a thickness of (40 ± 7) nm of the thin film. (Scale bar: 5 µm)

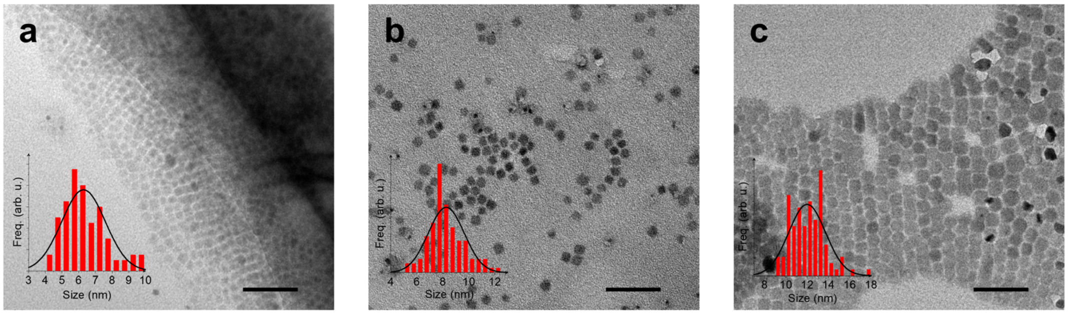

Supplementary Figure 6 Transmission electron microscopy characterization of $CsPbBr_3$ NCs. The TEM images of NCs were measured by a JEM-1400Flash electron microscope operated with an accelerating voltage of 120 kV. The sizes of NCs were estimated to be (6.3 ± 1.3) nm **a**, (8.3 ± 1.4) nm **b**, and (12.0 ± 1.7) nm **c**, respectively. These samples are termed NC6, NC8 and NC12, respectively. (All scale bars are 50 nm)

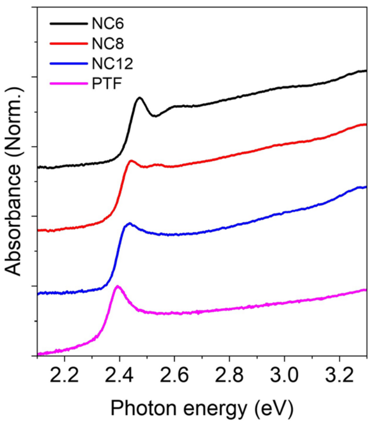

Supplementary Figure 7 Linear absorption spectra of NC6, NC8, NC12 and PTF at RT.

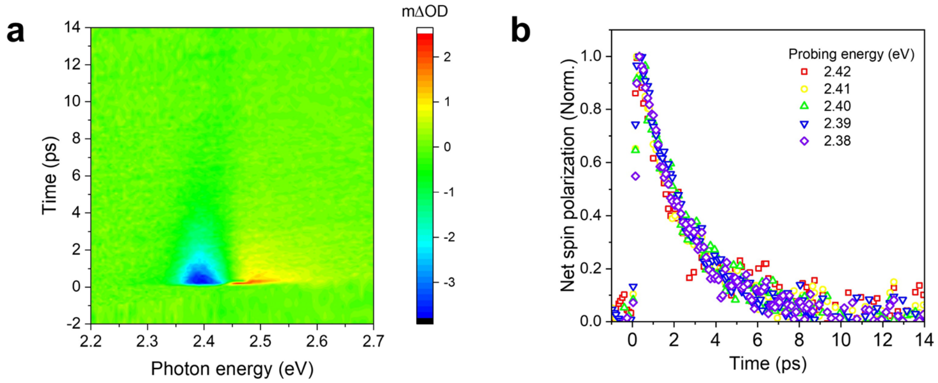

Supplementary Figure 8 Comparison of the spin-relaxation time at different probing energies at RT. a Subtracted CTA map of NC8 at RT. **b** Selected net spin signals at different probe energies. As mentioned in the Main text, the net spin polarization is extracted by taking the difference between the CTA results from co-circular and counter-circular configurations. Figure S8a shows the subtracted signals from a NC8 sample, and a bleaching signal centered slightly below the bandgap is observed. Since the biexciton state $|+1\rangle|-1\rangle$ share the population dynamics with the excited $|+1\rangle$ state and the photoinduced state superpose with the spin-independent components[19, 20], we probe the maximum bleaching near the biexciton resonance to extract the spin-relaxation time where the same net spin dynamics are acquired probing at different energies as shown in **b**.

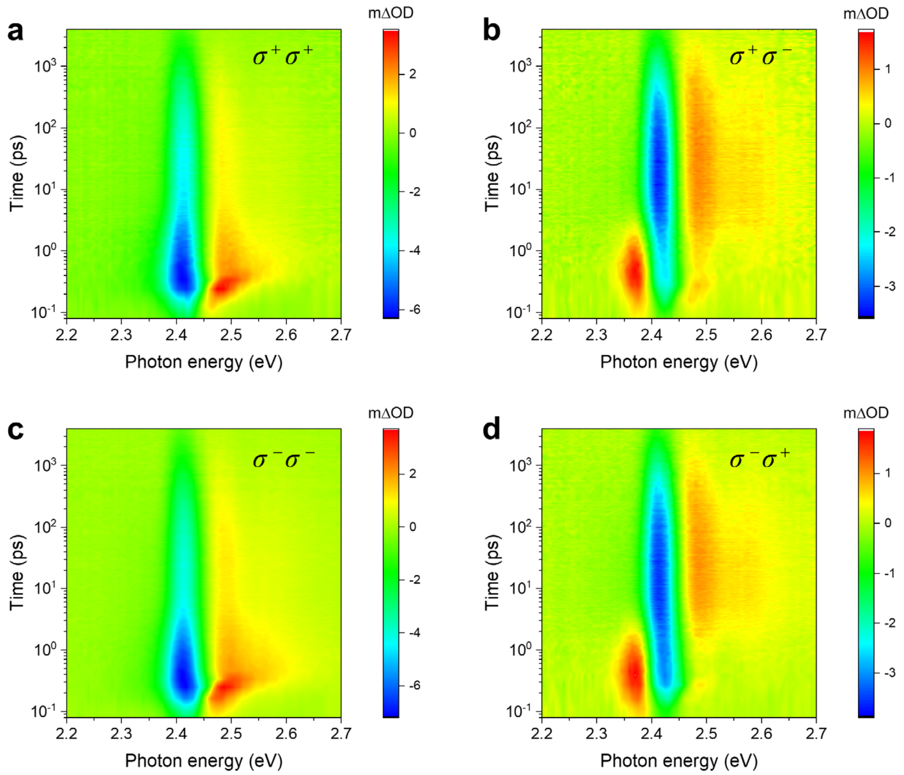

Supplementary Figure 9 Optical orientation of excitons in $CsPbBr_3$ NCs at RT. Circularly-polarized transient absorption measurements of NC8 (<*N*> ~ 0.3) with a configuration of $\sigma^+\sigma^+$ **a**, $\sigma^+ \sigma^-$ **b**, $\sigma^-\sigma^-$ **c**, and $\sigma^-\sigma^+$ **d**. The optical orientation of excitons here is verified by two sets of co/counter-circular CTA measurements, *i.e.*, the σ^+ (σ^-) photons-generated $|+1\rangle$ ($|-1\rangle$) excitons are probed by σ^+ (σ^-) and σ^- (σ^+) photons. The co-circular configurations $\sigma^+\sigma^+$ and $\sigma^-\sigma^-$ (counter-circular configurations $\sigma^+ \sigma^-$ and $\sigma^-\sigma^+$) are expected to give the same CTA results as shown in Figure S9a and S9c (S9b and S9d). The photo-induced absorption at ~2.45 eV during the first hundreds of femtoseconds in $\sigma^+\sigma^+$ and $\sigma^-\sigma^-$ configurations is probably due to the optical Stark effect[21] or four-wave mixed signal[22] which can be clearly resolved after subtraction as shown in Supplementary Fig. 8a.

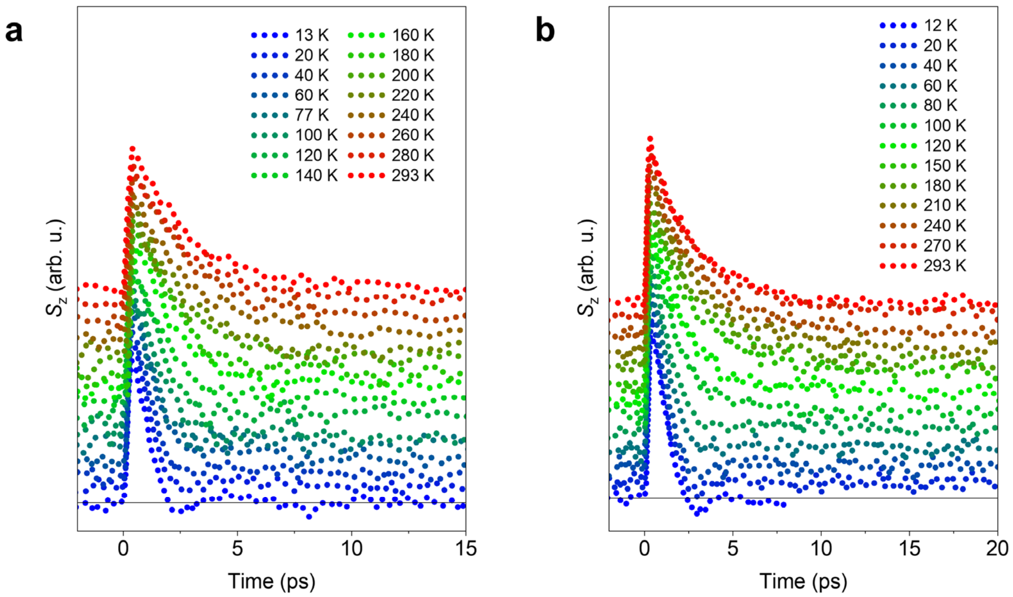

Supplementary Figure 10 Temperature-dependent net-spin polarization signals for $CsPbBr_3$ NC8 **a**, and NC12 **b**.

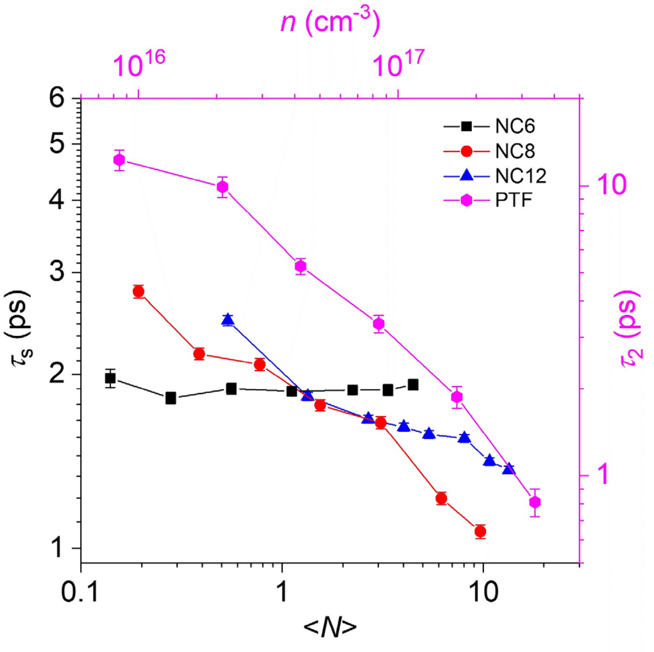

Supplementary Figure 11 The effect of pump fluence on the spin lifetime at RT. The exciton-exciton collisions may play an important role in spin relaxation at elevated densities. Accordingly, with a given temperature, the spin lifetime is expected to decrease with excitation density for the EY process while increase due to the motional narrowing process. However, both PTF and NCs exhibit enhanced spin-relaxation rates with increasing densities except for the strongly confined NC6 which shows an extremely weak dependence at RT. In general, the spin-relaxation time is longer for PTF than that for NCs indicating less effective spin depolarization in PTF. With the carrier density ranging from ~10^{16} to ~10^{17} cm^{-3}, τ_2 decreases significantly with excitation density suggesting the enhanced multiparticle scattering enhances the EY process. Similar tendencies are observed for NC8 and NC12, which can be explained by the dominating EY process at RT. Here, the average exciton occupation number <N> for NCs is estimated by fluence-dependent pump-probe measurements[23].

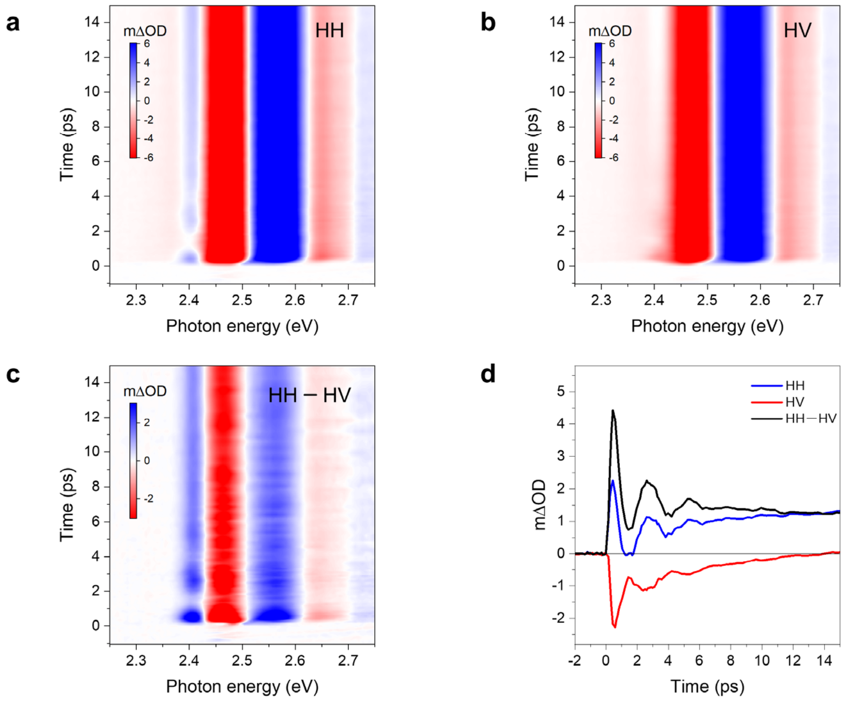

Supplementary Figure 12 Exciton quantum beating in $CsPbBr_3$ NCs observed by linearly-polarized pump and probe at 12 K. a TA spectra measured by horizontal pump and horizontal probe. **b** TA spectra measured by horizontal pump and vertical probe. **c** Exciton quantum beating map by linearly-polarized measurements. **d** The oscillatory dynamics around biexciton resonance for different pump-probe configurations.

References

1. Henzler, P. et al. Femtosecond Transfer and Manipulation of Persistent Hot-Trion Coherence in a Single CdSe/ZnSe Quantum Dot. *Phys. Rev. Lett.* **126**, 067402 (2021).
2. Mukamel, S. Principles of Nonlinear Optical Spectroscopy. (Oxford University Press, 1995).
3. Pikus, G.E. & Titkov, A.N. in Modern Problems in Condensed Matter Sciences, Vol. 8. (eds. F. Meier & B.P. Zakharchenya) 73-131 (Elsevier, 1984).
4. Strohmair, S. et al. Spin Polarization Dynamics of Free Charge Carriers in CsPbI3 Nanocrystals. *Nano Lett.* **20**, 4724-4730 (2020).
5. Karakus, M. et al. Phonon-Electron Scattering Limits Free Charge Mobility in Methylammonium Lead Iodide Perovskites. *J. Phys. Chem. Lett.* **6**, 4991-4996 (2015).
6. Wright, A.D. et al. Electron-phonon coupling in hybrid lead halide perovskites. *Nat. Commun.* **7** (2016).
7. Iaru, C.M., Geuchies, J.J., Koenraad, P.M., Vanmaekelbergh, D. & Silov, A.Y. Strong Carrier-Phonon Coupling in Lead Halide Perovskite Nanocrystals. *ACS Nano* **11**, 11024-11030 (2017).
8. Zhao, W. et al. Transient circular dichroism and exciton spin dynamics in all-inorganic halide perovskites. *Nat. Commun.* **11**, 5665 (2020).
9. Zhou, M., Sarmiento, J.S., Fei, C., Zhang, X. & Wang, H. Effect of Composition on the Spin Relaxation of Lead Halide Perovskites. *J. Phys. Chem. Lett.* **11**, 1502-1507 (2020).
10. Hirotsu, S., Harada, J., Iizumi, M. & Gesi, K. Structural Phase Transitions in CsPbBr3. *J. Phys. Soc. Jpn.* **37**, 1393-1398 (1974).
11. Chen, X., Lu, H., Yang, Y. & Beard, M.C. Excitonic Effects in Methylammonium Lead Halide Perovskites. *J. Phys. Chem. Lett.* **9**, 2595-2603 (2018).
12. Guo, Y. et al. Dynamic emission Stokes shift and liquid-like dielectric solvation of band edge carriers in lead-halide perovskites. *Nat. Commun.* **10**, 1175 (2019).
13. Žutić, I., Fabian, J. & Das Sarma, S. Spintronics: Fundamentals and applications. *Rev. Mod. Phys.* **76**, 323-410 (2004).
14. Odenthal, P. et al. Spin-polarized exciton quantum beating in hybrid organic–inorganic perovskites. *Nat. Phys.* **13**, 894-899 (2017).
15. Paul, G., Chatterjee, S., Bhunia, H. & Pal, A.J. Self-Doping in Hybrid Halide Perovskites via Precursor Stoichiometry: To Probe the Type of Conductivity through Scanning Tunneling Spectroscopy. *J. Phys. Chem. C* **122**, 20194-20199 (2018).
16. Shi, T., Yin, W.-J., Hong, F., Zhu, K. & Yan, Y. Unipolar self-doping behavior in perovskite CH3NH3PbBr3. *Appl. Phys. Lett.* **106**, 103902 (2015).
17. Feldmann, S. et al. Photodoping through local charge carrier accumulation in alloyed hybrid perovskites for highly efficient luminescence. *Nat. Photonics* **14**, 123-128 (2019).
18. Wang, H. et al. Trifluoroacetate induced small-grained CsPbBr3 perovskite films result in efficient and stable light-emitting devices. *Nat. Commun.* **10**, 665 (2019).

19. Tao, W., Zhou, Q. & Zhu, H. Dynamic polaronic screening for anomalous exciton spin relaxation in two-dimensional lead halide perovskites. *Sci. Adv.* **6** (2020).
20. Giovanni, D. et al. Tunable room-temperature spin-selective optical Stark effect in solution-processed layered halide perovskites. *Sci. Adv.* **2**, e1600477 (2016).
21. Cunningham, P.D., Hanbicki, A.T., Reinecke, T.L., McCreary, K.M. & Jonker, B.T. Resonant optical Stark effect in monolayer WS2. *Nat. Commun.* **10**, 5539 (2019).
22. Trifonov, A.V. et al. Multiple-frequency quantum beats of quantum confined exciton states. *Phys. Rev. B* **92** (2015).
23. Mondal, A. et al. Ultrafast exciton many-body interactions and hot-phonon bottleneck in colloidal cesium lead halide perovskite nanocrystals. *Phys. Rev. B* **98** (2018).
